# Emotion-related theories in classroom language learning: the conceptualization and causation of emotions

**DOI:** 10.3389/fpsyg.2025.1551640

**Published:** 2025-02-26

**Authors:** Wenjie Wu, Muhammad Kamarul Kabilan

**Affiliations:** ^1^School of Educational Studies, University Sains Malaysia, Penang, Malaysia; ^2^Faculty of Letters, Universitas Negeri Malang, East Java, Indonesia

**Keywords:** emotions, theoretical approaches, SLA, conceptualization, causation

## Abstract

Language classrooms are embedded with a wide range of emotions. Emotions play a significant role in affecting learners' language learning and academic performance. Yet, while the role of emotions in L2 classrooms has been recognized, very scant studies have investigated the underlying theoretical frameworks in great depth with regard to the conceptualization and causation of emotions. Moreover, very few review studies have paid sufficient attention to the antecedents or causes of emotions underpinned by certain theories in the field of SLA. Therefore, to offer a complementary review of emotion-related theories and provide fresh insights into the emotional research in SLA, the present study first explains the theoretical approaches of the conceptualization and causation of emotions, elucidates how these theories are applied into the emotional research in language learning, and identifies the effect of the interplay between cognitive, psychological, social, and contextual factors on the emotional development in the language learning. Finally, practical implications, like emotional regulation strategies for both language teachers and learners and future directions, like the integration with AI tools for L2 researchers, language teachers, and teacher educators who are interested in emotional research are also discussed.

## 1 Introduction

Emotions have emerged as one of the most significant issues in contemporary educational research (Dewaele, 2021). For a few decades, educational research has put a strong focus on the cognitive development of learning and teaching and neglected emotions until the 1990s, when there was an affective transition in educational science (Li, 2020). Researchers in the field of education started to recognize the fact that emotions are prevalent in classroom contexts. Also, they began to realize the fact that teachers and students frequently experience a rich variety of emotions that are complex, manifold, and momentous (Schutz and Pekrun, 2007). In view of this, emotions are no longer regarded as irrelevant phenomena occurring in educational settings. Instead, it is acknowledged that emotions should be considered as greatly significant for students' learning, psychological wellbeing, and all-round development. In addition, emotions can also affect teachers' professional development and classroom instructions, which could have great influence in the productivity of schools and other educational institutions all over the world (Pekrun and Linnenbrink-Garcia, 2014).

In the area of language learning, language classrooms are prevalent with a wide range of emotions, such as anxiety, enjoyment, guilt, and boredom (Bown and White, 2010; Swain, 2013). Emotions are considered to have an impact on second language (L2) learners' learning and performance. They do so by guiding the attentional processes of L2 learners and the utilization of cognitive resources, arousing and maintaining learners' interest in language learning materials, activating various modes of information processing, and either facilitating or impeding learners' engagement and self-regulation in the learning process (Pekrun, 2006; Schumann, 1994). Given the significant role of emotions in second language acquisition (SLA), there has been an increasing number of studies on emotions in SLA over the past three decades (Saito et al., 2018). Nevertheless, although some L2 researchers have acknowledged the vital role of emotions in language learning and conducted a substantial amount of research on emotions, with a primary focus on negative emotions, particularly L2 anxiety (Cheng, 2017; Dewaele et al., 2008), emotions still remain underresearched, which is especially evident when contrasted with the extensive exploration of mainstream cognitive development in language learning studies (Shao et al., 2019; Li et al., 2024).

Despite the important role that emotions play in learners' language learning process, the prior studies have mostly investigated negative emotions and the relationships between emotions and other variables (e.g., Alrabai, 2022; Wang et al., 2021). Very few L2 researchers have paid attention to the application of emotion theories underpinning the conceptualization and causation of emotions (Dewaele and Li, 2020). Furthermore, there have been very scant review studies aimed at elucidating the emotional theories and shedding light on their distinctive characteristics. For example, Wang (2024) specifically reviewed how positive psychology (PP) was developed in the study of emotions in SLA from its theoretical foundations, study topics, and study methods. Moreover, Oxford (2015) synthesized some theories of emotion with relation to language learning, such as PP, social psychology, social constructivism, and social constructionism. Noels et al. (2019) particularly evaluated how self-determination theory (SDT) was applied to understanding the development of language learners' motivational orientations and engagement. In spite of the prior works on emotional theories, there is still much more work to do. Therefore, the present study seeks to delineate the underlying theoretical paradigms of the conceptualization and causation of emotions, demonstrate the applications and contributions of these theories pertinent to emotional research in SLA, and provide practical implications and directions for L2 researchers and language teachers. More specifically, the study presents research findings that identify the causal links between emotions and other factors, which may help L2 researchers gain some novel insights and find new directions in emotions for future empirical research.

## 2 Emotional theories in SLA

The emotional research in the field of language learning can be traced back to the 1970s. During the period of the early 1960s and the mid-1980s, the research attention in SLA was predominantly focused on the cognitive factors (Prior, 2019), which was known as the Emotion Avoidance Phase (Dewaele and Li, 2020). Later, between the mid-1980s and the early 2010s, the emotional research experienced the second phase where emotions started to gain more research attention, which was known as the anxiety-prevailing phase, with the dominant focus on negative emotions (MacIntyre, 2017). Since the early 2010, when positive psychology was introduced into applied linguistics (MacIntyre, 2016), and emotional research in SLA came into the third phase, “the Positive and Negative Emotion Phase.” Despite a bulk of emotion studies published in SLA over the past few decades, L2 researchers pay little scholarly attention to illuminate the concept of emotions and elucidate the underpinning theories on the conceptualization of emotions in L2 learning (Dewaele and Li, 2020). In terms of the conceptualization of L2 emotions, two approaches are involved: the basic approach and the dimensional approach (Dewaele and Li, 2020). Based on the basic emotional theory, the fundamental proposition can be traced back to the traditional assumptions proposed by Ekman (1984), who contended that human emotions include several basic emotions. These emotions, including six basic ones (i.e., happy, surprise, fear, disgust, angry, and sad), are characterized as discrete, universal, and biologically driven, as well as linked with specific behavioral and facial expressions and action tendencies. By contrast, according to the dimensional theory, emotional constructs are perceived as including three independent dimensions: pleasure/valence, arousal/activation, and dominance/control, which can be found in the “PAD Model” (Russell and Mehrabian, 1974).

In terms of the early emotional research in SLA, some have adopted the Affective Filter Hypothesis (Krashen, 1985), which posited that four affective factors, including motivation, attitudes, self-confidence, and anxiety, influence how effectively language input is processed and absorbed by screening input language. Krashen's (1985) main viewpoints entail that an increased affective filter can inhibit input, whereas a lowered affective filter can promote the language input to be acquired (Du, 2009). Moreover, the affective filter plays a crucial role in accounting for the individual differences in the process of SLA and language learning. This theory has laid the theoretical foundation for many empirical studies in SLA (e.g., Rahman et al., 2019; Chen, 2020). Although this theory has made much contribution to explaining the potential effects of emotions on learners' language acquisition, the affective filter has been criticized for its limitations in L2 emotional research, like oversimplication of the emotional factors and underlying mechanisms and overemphasis on language input. Meanwhile, in the stages of the 70s and 80s, learner beliefs have been the main concern of researchers in the field of applied linguistics (AL). Given the inseparable relationship between cognition and emotion as recognized in AL (Gieve and Miller, 2006; Pavlenko, 2005) and the important role of identity in AL (Block, 2007), there was a crucial need to understand the interrelationship between beliefs, identities, and emotions. For example, Barcelos (2015) has elaborated on the reciprocal relationship between beliefs, identities, and emotions and concluded that they were intrinsically and interactively related.

In addition, with the socio-cultural turn in the domain of SLA in the past two decades (Johnson, 2006), it also contributed to the shift of emotional research from the individual emotional development to the interaction between individual and social factors. One of the representative theories is the Socio-Cultural Theory (SCT) (Vygotsky, 1987), which posits that there is not only the development of cognition but also the generation of emotions when engaged in human activities. Compared to the traditional psychology that separated the emotions and cognitions, SCT highlights viewing learners' emotional experiences from a holistic perspective (Qin et al., 2022). In terms of the emotional experience, some scholars put the emphasis on the features of emotions, with the focus on the types of emotions and their development (e.g., Mok, 2015), whereas other academics perceived the emotional experience as the interplay of affect and cognition, showing the complex and comprehensive characteristics (e.g., Fleer et al., 2017; Qin et al., 2019). Similarly, social constructivism shares some similarities with SCT, which emphasizes that emotions are socially constructed and that language is crucial in developing and expressing the emotions. Panayiotou (2006) once contended that every language contains its own way of describing the world, including its own uses of emotion words. This constructs the way people in that culture experience emotions (Oxford, 2015). In other words, emotions that seem important in some cultures may not be linguistically existent in others.

Later, with the particular affective shift to positive emotions in the early 2010s, the flourishing development of Positive Psychology (PP) in SLA has brought novel theoretical approaches to theorize emotional causation in empirical studies. One major theory of PP is the Broaden-and-Build Theory (BBT), which addresses the significant effect of emotions. According to the Broaden-and-Build Theory, positive emotions tend to contribute to expansive thinking that broadens an individual's attention, cognition, and action, whereas negative emotions more likely narrow a person's scope of thinking and consciousness. More specifically, Fredrickson (2013) elaborated on the ways positive emotions function: (a) broaden thought-action repertoires, (b) undo the lingering effect of negative emotions, (c) boost psychological resiliency, (d) build personal resources, and (e) promote psychological and physical wellbeing. The BBT highlights the fundamental role of positive emotions, which have been marginalized in traditional psychological research, in affecting learners' learning and performance. Also, it differentiates the divergent functions and effects of both positive and negative emotions, which may provide a holistic lens for empirical studies (Fredrickson, 2001). Moreover, BBT points to the interplay between positive and negative emotions, which may serve as a theoretical underpinning in the emotional interaction research.

In addition to BBT, another basic theory from PP is concerned with the “EMPATHICS” model of wellbeing (Oxford, 2016). This theoretical model was developed from the “PERMA” model (Seligman, 2011), which represents positive emotions, engagement, relationship, meaning, and accomplishment. Later, Oxford (2016) expanded it into a nine-dimension framework. In terms of the nine-dimension model, Oxford (2016) emphasized that it does not intend to present a taxonomy. Instead, Oxford (2016) supported adopting a CDST perspective to examine the complex and holistic relationships between various dimensions with much attention to the social and cultural context. Although this theoretical model has rarely been used in exploring learners' emotions, it highlighted the non-linguistical goals in language learning, which advanced the traditional research in SLA with a mere focus on linguistic goals. What's more, emotional intelligence (EI), as one of the core concepts of the “EMPATHICS” model (Oxford, 2016), has gained extensive attention, particularly in the field of PP, whose ultimate goal is to facilitate human survival and success and improve human wellbeing (Allen et al., 2014). The theoretical framework of EI includes four emotion-related abilities: (1) perceiving and evaluating emotions; (2) accessing and expressing positive emotions; (3) understanding own and others' emotions; (4) managing and regulating own and others' emotions (Mayer and Salovey, 1997). Later, Petrides and Furnham (2001) differentiated two types of EI: trait EI and ability EI, with the former focusing on self-perceived personality traits on emotions and the latter highlighting one's situated and actual emotion-related competence. A number of empirical studies have confirmed the theoretical links between EI and emotions (e.g., Dewaele, 2013; Yu et al., 2015). Nonetheless, there is still a lack of research addressing the relations between EI and positive and negative emotions.

As for the emotional effect, the cognitive-motivational model of emotion effects (Pekrun, 2006) proposes that the effects of emotions on learners' L2 learning and performance are determined by the interaction with cognition and motivation, such as learners' learning strategies and motivational regulation. This model highlights how emotions, influenced by the interconnection with cognitive and motivational processes, play a crucial role in directing learners' thought and guiding their behaviors. More specifically, emotions can facilitate the adoption of various learning strategies, ranging from flexible approaches like elaborating on learning materials to more rigid methods such as basic rehearsal. Emotions can also drive distinct regulatory approaches, encompassing both autonomous self-regulation and externally facilitated regulation of learning processes (Pekrun and Perry, 2014). Moreover, emotions are believed to influence students' intrinsic motivation, driven by their interest and curiosity in learning, as well as their extrinsic motivation, which focuses on achieving desirable outcomes or avoiding undesirable ones. Besides, in terms of the relation between emotions and motivations, the L2 Motivational Self System (MSS), proposed by Dörnyei (2005), has also provided a theoretical framework to explore the interrelation between emotion and L2 motivations. As the discrepancies within the L2 self-conceptions, as sources of motivation, can lead to different emotional states, the L2 MSS can not only shed light on L2 learners' motivation but also provide insights into the causation of emotions (Papi, 2010).

Another theory that contributes to the conceptualization and causation of emotion is the Control-Value Theory (CVT). Under the CVT, emotions are conceptualized as achievement emotions, referring to emotions that are directly associated with achievement activities or outcomes in educational contexts (Pekrun, 2006; Pekrun and Perry, 2014). CVT highlights that the appraisals of control and value in achievement settings are the most proximal determinants of L2 learners' emotions. Based on the control-value theory, a three-dimensional taxonomy is classified according to valence (positive or negative quality), control (the degree of controllability), and object focus (activity or outcome). The most distinctive feature of CVT in conceptualizing emotions is that it expands the traditional perspectives, such as expectancy-value theories (Pekrun, 1992) and attributional theories of emotions (Weiner, 1985), by integrating these theoretical approaches for analyzing the diverse achievement emotions and addressing these emotions from a holistic perspective. According to the CVT, learners' perceived controllability and value of academic activities or outcomes are presumed to affect their achievement emotions. Specifically, when students have a high level of control and high positive value over the activities or outcomes, they will experience positive emotions, whereas when students feel a low level of control and high negative value, they may experience negative emotions (Pekrun et al., 2017). From the aspect of emotion causations, CVT highlights distinguishing the distinctive functions of diverse achievement emotions in affecting learners' cognitive, social, and psychological processes. More importantly, CVT classifies achievement emotions based on three dimensions, which take the temporal features of emotions into consideration by categorizing them into prospective, retrospective, and concurrent emotions. Also, CVT is unique in that it addresses the reciprocity and bidirectionality of emotions, which indicates that CVT can serve as a theoretical foundation for a broader range of empirical research, positioning emotions as both dependent and independent variables. However, CVT is not without limitations. It exclusively focuses on achievement-related emotions, which may not offer a holistic perspective of learners' various types of emotions. Given the wide range of emotions in language classrooms, emotions in SLA extend far beyond the domain of achievement emotions. Emotions with relation to “the contents of learning and teaching, to the process of cognitively generating knowledge, and to social interactions in the classroom” are equally important (Pekrun and Linnenbrink-Garcia, 2014, p. 3). Hence, it suggests that according to the three-dimensional taxonomy, there are at least four distinct groups of emotions that derserve more attention: achievement emotions, topic emotions, epistemic emotions, and social emotions (Pekrun et al., 2023). However, only a very few types of emotions, such as anxiety, enjoyment, and boredom, have been extensively researched in SLA.

Very recently, the inquiries into emotions in SLA have evolved significantly by conceptualizing emotions as a complex dynamic system (Wang et al., 2024). This new conceptualization can find its root in the Complex Dynamic System Theory (CDST), which has increasingly gained substantial attention and interests from L2 researchers (e.g., Elahi Shirvan and Talebzadeh, 2018; Yu et al., 2022). Compared to the previous traditional approach that viewed emotions as a stable trait-like variable and measured emotions by using homogenous and nomothetic techniques like Likert questionnaires, the CDST approach views emotion as a situated and context-dependent variable and addresses the dynamicity and complexity of emotions. CDST represents an epistemological approach that emphasizes a holistic understanding of systems and posits that phenomena are not made up of merely isolated components but of interconnected networks where elements interact dynamically and evolve over time (Han et al., 2023). Grounded in this framework, the investigation into emotional variables in language learning has led to innovative research orientations. Focusing on the emotion cauations, researchers taking CDST as a starting point presume that the dynamics of emotions arise from the interplay between cognitive, social, contextual, and psychological factors, thus contributing to the variations of language learners' behaviors and performance (Wang et al., 2024). What makes this theory distinct is that CDST highlights viewing the causal relations of emotion from a complex and dynamic lens, which provides a more in-depth understanding of the nature of emotions. This novel theoretical framework has also shed insight on the innovative methodological approaches (see Hiver and Al-Hoorie, 2019 for a review) compatible with the dynamic characteristics of emotions, pointing out the future research directions for the investigation of emotions in SLA. For example, Wang et al. (2024) have proposed a novel interpretation of emotions by integrating both macro and micro perspectives through the experience sampling method and the idiodynamic method, respectively, aiming to provide a more detailed understanding of the dynamic interplay of emotions in language learning. Freeborn et al. (2023) has elucidated how network analysis can be used as a novel techinique to explore the complex systems in SLA by modeling the structural relation among the influencial factors.

## 3 Empirical studies of emotions in SLA

A large number of emotional studies in SLA have been conducted from different aspects of emotional research. One strand of emotional inquiry into the causal relationship between other variables is the current research focus. In recent years, the majority of emotional studies have investigated the relationships between emotions and other correlates, including psychological, social, and contextual factors, such as motivation (Tian and McCafferty, 2022), cognition and language performance (Ma, 2022), and L2 achievement (Li, 2020). For example, Shao et al. (2020) examined the relations between achievement emotion and foreign language (FL) performance based on the CVT. The findings reveal a positive relationship between positive emotions and FL performance and a negative relation between negative emotions and FL performance. This finding resonates with Li's (2020) study, which combines both the CVT and BBT to explore the relationship between L2 learners' emotions (anxiety and enjoyment) and language achievement. In addition, drawing on L2 MSS, Papi (2010) examined the relations between ideal L2 self, ought-to L2 self, L2 anxiety, and L2 learning experience, revealing that the ideal L2 self and the L2 learning experience lowered students' English anxiety, whereas the ought-to L2 self significantly increased their level of anxiety. Moreover, building on the SCT, López (2022) explored the development of foreign language teacher identity under the influence of learning experiences, emotions, and socio-cultural context.

In terms of the causes or antecedents of emotions, certain individual and contextual factors have been reported to account for the causations of emotions. For example, drawing on the Emotional Intelligence Framework and PP, Li (2020) examined the effect of trait EI on L2 classroom emotions and L2 achievement. The results showed a positive relation between EI and FLE and EI and L2 achievement, which is in line with the findings in Chen et al. (2024). Moreover, Yang et al. (2021) adopted the CVT to explore the antecedents of learners' achievement emotions. The findings reveal that both individual (e.g., self-regulation) and environmental antecedents (teacher factors and peer factors) can affect students' achievement emotions. On the other hand, compared to the CVT, which exclusively relies on the learner-perceived appraisals of control and value of the activities or outcomes, CDST emphasizes taking a comprehensive view by including cognitive, psychological, individual, and environmental factors to account for the emotional development. For example, Huynh (2021) adopted CDST to explore L2 anxiety in online language learning. The results found several factors that affected the dynamics of anxiety, such as learners' familiarity with the system, learner autonomy, teacher factor, and tasks.

## 4 Implications and directions for future studies

To conclude, the main goal of the present study was to provide a complementary review of emotion-related theories for emotional research in SLA. The theories centered on the conceptualization and causations of emotions in SLA were explained in detail. Specifically, emotions are conceptualized as a dynamic, non-linear and interconnected system under the CDST lens, which allows for a more in-depth understanding by combining “shorter-term and narrower-scope system (micro levels)” and “longer-term and broader-scope structures (macro level)” (Wang et al., 2024, p. 105). By tracking the fluctuating trajectories of emotions over time, it can represent their “own unique patterns of peaks and troughs” at different timepoints (Wang et al., 2024). On the other hand, by highlighting the importance of investigating the dynamic interplay of factors at every single moment, it can capture the emergent, interconnected, and complex nature of emotions by identifying how various cognitive, psychological and contextual factors interacts.

Moreover, the application and contributions of these theoretical frameworks were also illustrated with the support of empirical evidence. From the literature reviewed, it can be acknowledged that emotions in language learning show the properties of complexity and dynamics. Due to this, the effect of emotions on language learning and performance can be complex, depending on the types of emotions. Besides, the causes or antecedents of emotions can be dynamic and diverse, involving a rich variety of factors as well as the interplay between them. However, the influential factors seem to have not been adequately investigated nor sufficiently theorized. Therefore, it deserves further exploration in the future emotional research.

These findings can be thought-provoking for both language teachers and researchers in emotional research in SLA. It is suggested that language teachers should implement emotional interventions, such as attributional restraining (Hall et al., 2016), mindset intervention (Dong, 2022), and value induction (Harackiewicz and Priniski, 2018), to help promote students' perceptions of control and/or value of tasks. Language teachers are also advised to be aware of their emotions, behaviors, and teaching style in order to influence students' positive emotions through emotional contagion (Frenzel et al., 2018). Zhao and Wang (2024) have explored the causes and consequences of emotional exhaustion as well as provided a theoretical frameowrk on regulation strategies for EFL teachers, which might help teachers regulate their emotions through the preventive and responsive ways. On the other hand, researchers in L2 emotions may use and even combine the established theories of emotions to guide their empirical exploration. Meanwhile, they can expand the existing theories of emotions by considering emotions in diverse learning contexts, such as technology-enhanced classroom learning (Butler, 2017) and digital settings (Lee and Lee, 2020), as well as in the four specific skills in language learning, such as L2 listening (Wang and MacIntyre, 2021) and writing class (Ariyanti et al., 2023). Wu et al. (2024) have investigated the role of artificial intelligence (AI) in affecting EFL learners' emotional engagement and behavioral intention, which may shed light on future emotional research by integrating AI applications in language learning. Moreover, L2 researchers should also extend the research designs and methodological approaches compatible with the adopted theories in future research. When investigating the L2 emotions from the CDST lens, researchers should consider integrative designs by drawing both qualitative and quantitative methods, such as the idiodynamic method, to advance the knowledge in the dynamic and complex nature of L2 emotions in language learning (Hiver et al., 2022).

## References

[B1] Allen V. MacCann C. Matthews G. Roberts R. D. (2014). “Emotional intelligence in education: from pop to emerging science,” in International Handbook of Emotions in Education, eds. R. Pekrun and L. Linnenbrink-Garcia (New York, NY: Routledge), 162–182.

[B2] Alrabai F. (2022). Modeling the relationship between classroom emotions, motivation, and learner willingness to communicate in EFL: applying a holistic approach of positive psychology in SLA research. J. Multiling. Multicult. Dev. 43, 2465–2483. 10.1080/01434632.2022.2053138

[B3] Ariyanti A. Setiawan S. Munir A. (2023). Students' enjoyment and anxiety in reminiscing about mind-mapping use in the English writing class. Stud. Engl. Lang. Educ. 10, 789–804. 10.24815/siele.v10i2.28215

[B4] Barcelos A. M. F. (2015). Unveiling the relationship between language learning beliefs, emotions, and identities. Stud. Second Lang. Learn. Teach. 5, 301–325. 10.14746/ssllt.2015.5.2.6

[B5] Block D. (2007). Second Language Identities. New York, NY: Continuum.

[B6] Bown J. White C. J. (2010). Affect in a self-regulatory framework for language learning. System 38, 432–443. 10.1016/j.system.2010.03.016

[B7] Butler Y. G. (2017). Motivational elements of digital instructional games: a study of young L2 learners' game designs. Lang. Teach. Res. 21, 735–750. 10.1177/1362168816683560

[B8] Chen C. (2020). The application of affective filter hypothesis theory in English grammar teaching. J. Contemp. Educ. Res. 4, 71–74. 10.26689/jcer.v4i6.1294

[B9] Chen Z. Zhang P. Lin Y. Li Y. (2024). Interactions of trait emotional intelligence, foreign language anxiety, and foreign language enjoyment in the foreign language speaking classroom. J. Multiling. Multicult. Dev. 45, 374–394. 10.1080/01434632.2021.1890754

[B10] Cheng Y. S. (2017). Development and preliminary validation of four brief measures of L2 language-skill-specific anxiety. System 68, 15–25. 10.1016/j.system.2017.06.009

[B11] Dewaele J.-M. (2013). “Emotions and language learning,” in Routledge Encyclopedia of Language Teaching and Learning, 2nd Edn., eds. M. Byram and A. Hu (New York, NY: Routledge), 217–220.

[B12] Dewaele J.-M. Petrides K. V. Furnham A. (2008). Effects of trait emotional intelligence and sociobiographical variables on communicative anxiety and foreign language anxiety among adult multilinguals: a review and empirical investigation. Lang. Learn. 58, 911–960. 10.1111/j.1467-9922.2008.00482.x

[B13] Dewaele J. M. (2021). “Research on emotions in second language acquisition: reflections on its birth and unexpected growth,” in Researching Language Learning Motivation: A Concise Guide, eds. A. H. Al-Hoorie and F. Szabó (London: Bloomsbury Academic), 125–134. 10.5040/9781350166912.ch-12

[B14] Dewaele J. M. Li C. (2020). Emotions in second language acquisition: a critical review and research agenda. Foreign Lang. World 196, 34–49.

[B15] Dong L. (2022). Mindsets as predictors for Chinese young language learners' negative emotions in online language classes during the pandemic: mediating role of emotion regulation. J. Multiling. Multicult. Dev. 45, 1–16. 10.1080/01434632.2022.2159032

[B16] Dörnyei Z. (2005). The Psychology of the Language Learner: Individual Differences in Second Language Acquisition. Mahwah, NJ: Lawrence Erlbaum Associates.

[B17] Du X. (2009). The affective filter in second language teaching. Asian Soc. Sci. 5, 162–165. 10.5539/ass.v5n8p16239392866

[B18] Ekman P. (1984). “Expression and the nature of emotion,” in Approaches to Emotion, eds. K. R. Scherer and P. Ekman (Hillsdale, NJ: Lawrence Erlbaum Associates), 319–344.

[B19] Elahi Shirvan M. Talebzadeh N. (2018). Exploring the fluctuations of foreign language enjoyment in conversation: an idiodynamic perspective. J. Intercult. Commun. Res. 47, 21–37. 10.1080/17475759.2017.1400458

[B20] Fleer M. González Rey F. Veresov N. (eds.). (2017). Perezhivanie, Emotions, and Subjectivity: Advancing Vygotsky's Legacy. Singapore: Springer. 10.1007/978-981-10-4534-9

[B21] Fredrickson B. L. (2001). The role of positive emotions in positive psychology: the broaden-and-build theory of positive emotions. Am. Psychol. 56, 218–226. 10.1037/0003-066X.56.3.218 11315248 PMC3122271

[B22] Fredrickson B. L. (2013). “Positive emotions broaden and build,” in Advances in Experimental Social Psychology, Vol. 47, ed. J. M. Olson (San Diego, CA: Academic Press), 1–53. 10.1016/B978-0-12-407236-7.00001-2

[B23] Freeborn L. Andringa S. Lunansky G. Rispens J. (2023). Network analysis for modeling complex systems in SLA research. Stud. Second Lang. Acquis. 45, 526–557. 10.1017/S0272263122000407

[B24] Frenzel A. C. Becker-Kurz B. Pekrun R. Goetz T. Lüdtke O. (2018). Emotion transmission in the classroom revisited: a reciprocal effects model of teacher and student enjoyment. J. Educ. Psychol. 110, 628–639. 10.1037/edu0000228

[B25] Gieve S. Miller I. K. (eds.). (2006). Understanding the Language Classroom. London: Palgrave. 10.1057/9780230523166

[B26] Hall N. C. Sampasivam L. Muis K. R. Ranellucci J. (2016). Achievement goals and emotions: the mediational roles of perceived progress, control, and value. Br. J. Educ. Psychol. 86, 313–330. 10.1111/bjep.12108 26917420

[B27] Han Z. Kang E. Y. Sok S. (2023). The complexity of epistemology and ontology in second language acquisition: a critical review. Stud. Second Lang. Acquis. 45, 1388–1412. 10.1017/S0272263122000420

[B28] Harackiewicz J. M. Priniski S. J. (2018). Improving student outcomes in higher education: the science of targeted intervention. Annu. Rev. Psychol. 69, 409–435. 10.1146/annurev-psych-122216-011725 28934586 PMC6211287

[B29] Hiver P. Al-Hoorie A. H. (2019). Research Methods for Complexity Theory in Applied Linguistics, Vol. 137. Bristol: Multilingual Matters. 10.21832/978178892575437917319

[B30] Hiver P. Al-Hoorie A. H. Evans R. (2022). Complex dynamic systems theory in language learning: a scoping review of 25 years of research. Stud. Second Lang. Acquis. 44, 913–941. 10.1017/S0272263121000553

[B31] Huynh T. N. (2021). A complex dynamic systems approach to foreign language learners' anxiety in the emergency online language classrooms. Comput. Assist. Lang. Learn. Electr. J. 22, 200–229.

[B32] Johnson K. E. (2006). The sociocultural turn and its challenges for second language teacher education. TESOL Q. 40, 235–257. 10.2307/40264518

[B33] Krashen S. (1985). The Input Hypothesis: Issues and Implications. New York, NY: Longman.

[B34] Lee J. S. Lee K. (2020). Affective factors, virtual intercultural experiences, and L2 willingness to communicate in in-class, out-of-class, and digital settings. Lang. Teach. Res. 24, 813–833. 10.1177/1362168819831408

[B35] Li C. (2020). A positive psychology perspective on Chinese EFL students' trait emotional intelligence, foreign language enjoyment, and EFL learning achievement. J. Multiling. Multicult. Dev. 41, 246–263. 10.1080/01434632.2019.1614187

[B36] Li C. Li W. Jiang G. (2024). Emotions in second language learning: retrospect and prospect. Modern Foreign Lang. 47, 63–75.

[B37] López M. G. M. (2022). Emotions and sociocultural in foreign teacher identity. Int. J. Foreign Lang. 18, 1–16. 10.17345/rile18.3499

[B38] Ma Y. (2022). On the relationship between English as a foreign language learners' positive affectivity, academic disengagement, and communication apprehension. Front. Psychol. 12:828873. 10.3389/fpsyg.2021.828873 35242070 PMC8885619

[B39] MacIntyre P. D. (2016). “So far so good: an overview of positive psychology and its contributions to SLA,” in Positive Psychology Perspectives on Foreign Language Learning and Teaching, ed. D. Gałajda (Cham: Springer), 3–20. 10.1007/978-3-319-32954-3_1

[B40] MacIntyre P. D. (2017). “An overview of language anxiety research and trends in its development,” in New Insights into Language Anxiety: Theory, Research, and Educational Implications, eds. C. Gkonou, M. Daubney, and J.-M. Dewaele (Bristol: Multilingual Matters), 11–30. 10.2307/jj.22730706.5

[B41] Mayer J. D. Salovey P. (1997). “What is emotional intelligence?,” in Emotional Development and Emotional Intelligence: Implications for Educators, eds. P. Salovey and D. Sluyter (New York, NY: Basic Books), 3–31.

[B42] Mok N. (2015). Toward an understanding of perezhivanie for sociocultural SLA research. Lang. Sociocult. Theory 2, 139–159. 10.1558/lst.v2i2.26428

[B43] Noels K. A. Lou N. M. Vargas Lascano D. I. Chaffee K. E. Dincer A. Zhang Y. S. D.. (2019). “Self-determination and motivated engagement in language learning,” in The Palgrave Handbook of Motivation for Language Learning, eds. M. Lamb, K. Csizér, A. Henry, and S. Ryan (Cham: Springer International Publishing), 95–115. 10.1007/978-3-030-28380-3_5

[B44] Oxford R. (2015). Emotion as the amplifier and the primary motive: some theories of emotion with relevance to language learning. Stud. Second Lang. Learn. Teach. 7, 371–393. 10.14746/ssllt.2015.5.3.2

[B45] Oxford R. L. (2016). “Toward a psychology of well-being for language learners: the ‘EMPATHICS' vision,” in Positive Psychology in SLA, eds. T. Gregersen, P. D. MacIntyre, and S. Mercer (Bristol: Multilingual Matters), 10–87. 10.21832/9781783095360-003

[B46] Panayiotou A. (2006). “Translating guilt: an endeavor of shame in the Mediterranean?,” in Bilingual Minds: Emotional Experience, Expression, and Representation, ed. A. Pavlenko (Bristol: Multilingual Matters), 183–208. 10.21832/9781853598746-009

[B47] Papi M. (2010). The L2 motivational self system, L2 anxiety, and motivated behavior: a structural equation modeling approach. System 38, 467–479. 10.1016/j.system.2010.06.011

[B48] Pavlenko A. (2005). Emotions and Multilingualism. Cambridge: Cambridge University Press. 10.1017/CBO9780511584305

[B49] Pekrun R. (1992). The impact of emotions on learning and achievement: towards a theory of cognitive/motivational mediators. Appl. Psychol. 41, 359–376. 10.1111/j.1464-0597.1992.tb00712.x

[B50] Pekrun R. (2006). The control-value theory of achievement emotions: assumptions, corollaries, and implications for educational research and practice. Educ. Psychol. Rev. 18, 315–341. 10.1007/s10648-006-9029-9

[B51] Pekrun R. Lichtenfeld S. Marsh H. W. Murayama K. Goetz T. (2017). Achievement emotions and academic performance: longitudinal models of reciprocal effects. Child Dev. 88, 1653–1670. 10.1111/cdev.12704 28176309

[B52] Pekrun R. Linnenbrink-Garcia L. (2014). “Introduction to emotions in education,” in International Handbook of Emotions in Education, eds. R. Pekrun and L. Linnenbrink-Garcia (New York, NY: Routledge), 1–10. 10.4324/9780203148211

[B53] Pekrun R. Marsh H. W. Elliot A. J. Stockinger K. Perry R. P. Vogl E.. (2023). A three-dimensional taxonomy of achievement emotions. J. Pers. Soc. Psychol. 124:145. 10.1037/pspp0000448 36521161

[B54] Pekrun R. Perry R. P. (2014). “Control-value theory of achievement emotions,” in International Handbook of Emotions in Education, eds. R. Pekrun and L. Linnenbrink-Garcia (New York, NY: Routledge), 120–141.

[B55] Petrides K. V. Furnham A. (2001). Trait emotional intelligence: psychometric investigation with reference to established trait taxonomies. Euro. J. Pers. 15, 425–448. 10.1002/per.416

[B56] Prior M. T. (2019). Elephants in the room: an “affective turn,” or just feeling our way? Modern Lang. J. 103, 516–527. 10.1111/modl.12573

[B57] Qin L. L. He Y. H. Ouyang X. B. (2019). A narrative study on the influence of novice teachers' emotions on cognitive development. Modern Foreign Lang. 818–829.

[B58] Qin L. L. Yao L. Niu B. G. (2022). A critical review of L2 learners' emotions. Foreign Lang. Educ. China 5, 51–58, 92–93.

[B59] Rahman B. Hamid S. Gul A. (2019). Impact of stress on the performance of university students in the light of Krashen's affective filter theory. Liberal Arts Soc. Sci. Int. J. 3, 59–64. 10.47264/idea.lassij/3.2.7

[B60] Russell J. A. Mehrabian A. (1974). Distinguishing anger and anxiety in terms of emotional response factors. J. Consult. Clin. Psychol. 42, 79–83. 10.1037/h0035915 4814102

[B61] Saito K. Dewaele J. M. Abe M. In'nami Y. (2018). Motivation, emotion, learning experience, and second language comprehension development in classroom settings: a cross-sectional and longitudinal study. Lang. Learn. 68, 709–743. 10.1111/lang.12297

[B62] Schumann J. H. (1994). Where is cognition? Emotion and cognition in second language acquisition. Stud. Second Lang. Acquis. 16, 231–242. 10.1017/S0272263100012894

[B63] Schutz P. A. Pekrun R. (eds.). (2007). Emotion in Education. Boston, MA: Academic Press.

[B64] Seligman M. E. P. (2011). Flourish: A Visionary New Understanding of Happiness and Well-being. New York, NY: Atria/Simon & Schuster.

[B65] Shao K. Pekrun R. Marsh H. W. Loderer K. (2020). Control-value appraisals, achievement emotions, and foreign language performance: a latent interaction analysis. Learn. Instruct. 69:101356. 10.1016/j.learninstruc.2020.101356

[B66] Shao K. Pekrun R. Nicholson L. J. (2019). Emotions in classroom language learning: what can we learn from achievement emotion research? System 86:102121. 10.1016/j.system.2019.102121

[B67] Swain M. (2013). The inseparability of cognition and emotion in second language learning. Lang. Teach. 46, 195–207. 10.1017/S0261444811000486

[B68] Tian L. McCafferty S. G. (2022). The dynamic interaction of second language motivation and emotional experience: the case of Chinese learners of English. Front. Psychol. 13:905429. 10.3389/fpsyg.2022.905429 35734449 PMC9207525

[B69] Vygotsky L. S. (1987). The Collected Works of L. S. Vygotsky: Problems of the Theory and History of Psychology, Vol. 3. Springer Science & Business Media.

[B70] Wang H. Peng A. Patterson M. M. (2021). The roles of class social climate, language mindset, and emotions in predicting willingness to communicate in a foreign language. System 99:102529. 10.1016/j.system.2021.102529

[B71] Wang L. MacIntyre P. D. (2021). Second language listening comprehension: the role of anxiety and enjoyment in listening metacognitive awareness. Stud. Second Lang. Learn. Teach. 11, 491–515. 10.14746/ssllt.2021.11.4.2

[B72] Wang P. Ganushchak L. Welie C. van Steensel R. (2024). The dynamic nature of emotions in language learning contexts: theory, method, and analysis. Educ. Psychol. Rev. 36:105. 10.1007/s10648-024-09946-2

[B73] Wang Y. (2024). A review of emotions in second language acquisition from a positive psychology perspective. Int. J. Educ. Human. 4, 287–296. 10.58557/(ijeh).v4i3.241

[B74] Weiner B. (1985). An attributional theory of achievement motivation and emotion. Psychol. Rev. 92, 548–573. 10.1037/0033-295X.92.4.548 3903815

[B75] Wu H. Wang Y. Wang Y. (2024). To use or not to use? A mixed-methods study on the determinants of EFL college learners' behavioral intention to use AI in the distributed learning context. Int. Rev. Res. Open Distribut. Learn. 25, 158–178. 10.19173/irrodl.v25i3.7708

[B76] Yang Y. Gao Z. Han Y. (2021). Exploring Chinese EFL learners' achievement emotions and their antecedents in an online English learning environment. Front. Psychol. 12:722622. 10.3389/fpsyg.2021.722622 34721180 PMC8554019

[B77] Yu H. Peng H. Lowie W. M. (2022). Dynamics of language learning motivation and emotions: a parallel-process growth mixture modeling approach. Front. Psychol. 13:899400. 10.3389/fpsyg.2022.899400 35800938 PMC9253624

[B78] Yu W. H. Shao K. Q. Xiang Y. Z. (2015). The relationship between emotional intelligence, foreign language learning anxiety, and english learning achievement. Modern Foreign Lang. 656–666.

[B79] Zhao X. Wang Y. (2024). EFL teachers' perceptions of emotional exhaustion and associated regulation strategies: a phenomenological analysis. Innov. Lang. Learn. Teach. 18, 1–15. 10.1080/17501229.2024.2391377

